# Ecological specialization in vectors alters transmission thresholds and endemic dynamics in multi-host, multi-vector systems

**DOI:** 10.1371/journal.pcbi.1014754

**Published:** 2026-09-11

**Authors:** Benjamin M. Althouse

**Affiliations:** 1 Information School, University of Washington, Seattle, Washington, United States of America; 2 Department of Biology, New Mexico State University, Las Cruces, New Mexico, United States of America; University of California Irvine, UNITED STATES OF AMERICA

## Abstract

Modeling vector-borne pathogens that circulate among several host and vector species hinges on how the *force of infection* (FOI) is defined. In an *i*-host, *j*-vector susceptible-infectious-recovered modeling framework I compare two common FOI denominators: (i) a weighted form in which vectors bite preferred hosts disproportionately, and (ii) an unweighted form that assumes opportunistic biting. Using identical parameter sets calibrated to primate–*Aedes* data from Kédougou (Senegal), the weighted FOI doubles the basic reproduction number (*R*_0_) relative to the opportunistic FOI and can shift *R*_0_ across the epidemic threshold. It also produces higher long-run prevalence and larger oscillations. Both autonomous formulations undergo a forward transcritical bifurcation at *R*_0_ = 1. Selecting an ecologically realistic biting assumption is therefore critical for predicting sylvatic-to-urban spillover risk and designing interventions in multi-host systems.

## Author summary

Mosquito-borne diseases such as dengue and yellow fever circulate in forests where several mosquito and animal species interact. I wanted to know how much it matters which host a mosquito decides to bite. Using a model that follows a virus moving among two monkey species and two mosquito species, I tested two scenarios on either end of the behavioral range: mosquitoes that “home in” on their preferred host, and mosquitoes that bite whatever is most abundant.

Simply swapping this one behavioral assumption changed several model predictions. Targeted biting doubled the basic reproductive number in the baseline comparison, shifted the epidemic threshold, and produced higher long-run prevalence and larger oscillations. Opportunistic biting produced lower transmission and more strongly damped dynamics. Because these differences arise from the FOI formulation, field studies that measure real biting patterns are crucial for forecasting spillover into people and for deciding whether to focus on reducing certain hosts or controlling mosquito numbers.

By uniting ideas from biodiversity (“dilution effects”) and mosquito choice, my work offers practical guidance for researchers and health agencies working in multi-host disease systems.

## 1 Introduction

Pathogens exist in a world populated by many potential hosts and vectors. While evolution has directed many pathogens to specialize and infect a single or few hosts, other pathogens can infect a number of varied hosts and vectors [1–4]. Malaria [5], dengue virus [6–8], yellow fever virus [9], chikungunya virus [10,11], Rift Valley fever virus [12], West Nile virus [13], and Zika virus [7,11,14–17] all cause major morbidity and mortality worldwide and have transmission dynamics dictated by multiple host and vector species. While a recent systematic review reveals an accelerating shift toward multi-host formulations in West Nile Virus (WNV) [18], little consensus has been reached on the appropriate formulation of the host-vector model structure, and the majority of mathematical models describing vectored pathogen transmission focus on a single host and vector [19,20].

Many studies in disease ecology show that adding host species to a community can either amplify or reduce disease transmission [21]. This effect depends on each host’s competence and the vector’s feeding behavior. When host diversity reduces transmission, this is known as the dilution effect. Conversely, when additional hosts increase transmission – because they are highly competent or boost vector abundance – this is called amplification [22]. Alternate hosts can divert vectors from primary hosts (zooprophylaxis), or, if highly competent, they may increase transmission. These ecological dynamics underscore the importance of how the transmission term is represented in a multi-host disease model.

The force of infection (FOI), or transmission term, in mathematical models of disease dynamics dictates the rate of contact between susceptible and infectious hosts and vectors, incorporating the transmission potential of a pathogen [23]. Importantly, the transmission term feeds into the calculation of the basic reproductive number, *R*_0_, defined as the number of secondary cases caused by an infected individual in an entirely susceptible population [24]. It has been previously shown that different forms of the transmission term in host–vector models, often assumed to produce equivalent predictions, can give rise to conflicting predictions of disease dynamics [25]. In practice, this means that two models of the same system might reach opposite conclusions about whether an outbreak will occur, posing a challenge for public health planning.

Recent studies further highlight the importance of capturing host–vector heterogeneity. For example, Ross River virus (a multi-host, multi-vector arbovirus) is found in Australia, Papua New Guinea, and the islands of the South Pacific, and exhibits distinct transmission cycles driven by specific mosquito–host interactions. A 2021 analysis found that certain mosquito–bird pairs sustain an enzootic cycle of Ross River virus, whereas other mosquito–human pairs drive urban spillover, underscoring the role of particular host–vector combinations in transmission [26]. A growing body of literature has established that assumptions regarding disease transmission can generate conflicting predictions, and systematic reviews show that a significant fraction of models now deviate from classical mass-action assumptions to better capture ecological realities. Building on this, this work quantifies how innate vector feeding selectivity, when formalized in the force-of-infection term, changes transmission thresholds, long-run prevalence, and transient dynamics in a multi-host, multi-vector system. Similarly, theoretical work has shown that explicitly including vector host preference and host competence in a two-host model can determine whether adding a secondary host leads to a dilution or an amplification of disease risk [27]. These examples illustrate that host community composition and vector behavior can fundamentally shape pathogen spread.

At root, the two biting formulations I examine embody different causal narratives about what limits mosquito-host contact. The frequency-dependent (vector-limited) framework starts from the mosquito’s perspective: the pool of bites is fixed by mosquitoes’ physiological capacity for blood feeding, and hosts merely divide that finite resource among themselves. In contrast, the density-dependent (host-limited) framework is written from the host’s perspective: each host presents an independent “surface” that can, in principle, receive an unlimited number of probing attempts, so aggregate biting scales with the sum of host availabilities unless an explicit denominator is imposed. Normalizing the density-dependent expression by the weighted host total makes the two equations algebraically identical, but it does not erase this conceptual distinction: the first treats mosquito search effort as the bottleneck, the second treats host encounter probability as the bottleneck. Because the biological processes that control transmission (e.g., resting-site limitation versus host defensive behavior) differ between systems, the choice of framework is not merely a matter of prey specificity: it encodes our assumption about which side of the interaction is limiting and therefore dictates how new data (such as changes in vector abundance, host diversity, or feeding interruption rates) should be mapped onto model parameters.

In the following sections, I use a general modeling framework to examine two contrasting assumptions about mosquito host choice and their epidemiological consequences. I demonstrate how differences in multi-host, multi-vector pathogen model transmission terms correspond to distinct biological scenarios and lead to conflicting epidemiological predictions.

## 2 Methods and results

### 2.1 Mosquito host choice

There remains an open question whether vectors have strong innate preferences for particular hosts, or alternatively if vectors merely feed on whichever hosts are nearby and available. Several factors influence mosquito behavior, including temperature, humidity, and odors [28], which can guide host seeking and potentially create apparent host preferences. Carbon dioxide mediates both long- and short-range host seeking: long-range attraction is driven by CO_2_ and other organic molecules emitted from host skin, breath, and excreta [29]. Mosquito genetics may also influence individual host choice, as host attraction varies across vector and host species [30]. Environmental conditions, including temperature and humidity, can further modulate mosquito activity and host-seeking behavior [31].

Several studies have indicated the importance of host characteristics in mosquito host choice. For example, anthropometry is associated with more frequent biting for both *Anopheles* [32] and *Aedes* mosquitoes [33]. Additionally, host defensive behavior can play a role in vector choice. Chickens and house sparrows exhibit defensive behaviors against *Culex pipiens pipiens* mosquitoes, leading to fewer successful feeding attempts [34]. Inverse relationships between host defensiveness and mosquito feeding success have been observed for *Culex* [35] and *Aedes* mosquitoes [31,36,37]. Indeed, *Aedes* are well known to prefer human bloodmeals [38], and defenses such as screened windows and air conditioning are effective at reducing diseases like dengue [39] by limiting mosquito–human contact. Finally, field and modeling work now links temperature‑driven shifts in host‑feeding to epidemic potential [40].

Ultimately, identifying and generalizing mosquito host choice is challenging due to limitations in bloodmeal identification, environmental heterogeneities affecting host–vector encounters, and underlying genetic differences. Field work on *Aedes* in Panamá and Brazil now shows that the *realized* blood-meal spectrum contracts or expands with temperature-driven changes in host metabolic rate and CO_2_ output, amplifying early-season transmission bursts when primates are most attractive [41]. Complementary phase-type models of the entire bite sequence reveal that even small increases in *probing* or *ingestion* failure – caused, for example, by vigorous host defense – can offset higher host-seeking rates and lower the mosquito’s contribution to *R*_0_ [42]. Taken together with the temperature–feeding link already noted above, these studies underline that host choice is both *plastic* and *multi-stage*, reinforcing the need to match the FOI denominator to empirical feeding data rather than assuming a fixed preference matrix.

Recent experimental work with *Aedes albopictus* demonstrates that this vector *does not* feed preferentially on viremic hosts; in head-to-head trials the proportion of mosquitoes engorging on cynomolgus macaques did not differ from that on squirrel monkeys, and virus acquisition was negligible in both cases [4]. These findings caution against assuming that a vector’s innate arbovirus susceptibility correlates with its realized host preference matrix, further justifying a data-driven – rather than taxonomically inferred – parameterization of biting rates. Moreover, bite-challenge infections in cynomolgus and squirrel monkeys reveal that the duration–magnitude trade-off of viremia differs sharply between sylvatic DENV-2 and ZIKV, with the latter achieving shorter but higher peaks that translate into superior transmission to mosquitoes [7]. These host-specific within-host kinetics reinforce the need to couple host competence with empirically measured feeding preferences in multihost FOI formulations.

In summary, evidence supports the existence of both highly selective mosquito feeding (e.g., anthropophilic vectors targeting humans) and more opportunistic feeding driven by host availability. Therefore, one can conceive of a spectrum of possible host-seeking modes. In the next section, I incorporate these two extreme scenarios into a multi-host, multi-vector SIR model by altering the formulation of the force of infection to represent an “efficient” (targeted) vs. “inefficient” (opportunistic) vector. Notably, I assume host choice is entirely exogenous (driven by ecology); although some pathogens can influence host attractiveness or vector behavior (for instance, malaria parasites can increase mosquitoes’ attraction to infected hosts), such effects are beyond the scope of our model.

### 2.2 Modeling host-vector transmission

Many forms of the transmission term in multi-host, multi-vector models have been proposed. Building off a previous comprehensive review of host–vector models [20], I identified two predominant formulations of the transmission term, each corresponding to an extreme of mosquito host-seeking behavior: one in which mosquitoes home in on preferred hosts with high efficiency [43–48], and another in which mosquitoes may have innate preferences but are often “confused” by the cues of other host species (e.g., carbon dioxide, body odors, heat) and end up feeding on whatever host they first encounter [49–54]. There is no clear consensus on which formulation is more realistic [19].

**Fig 1:**
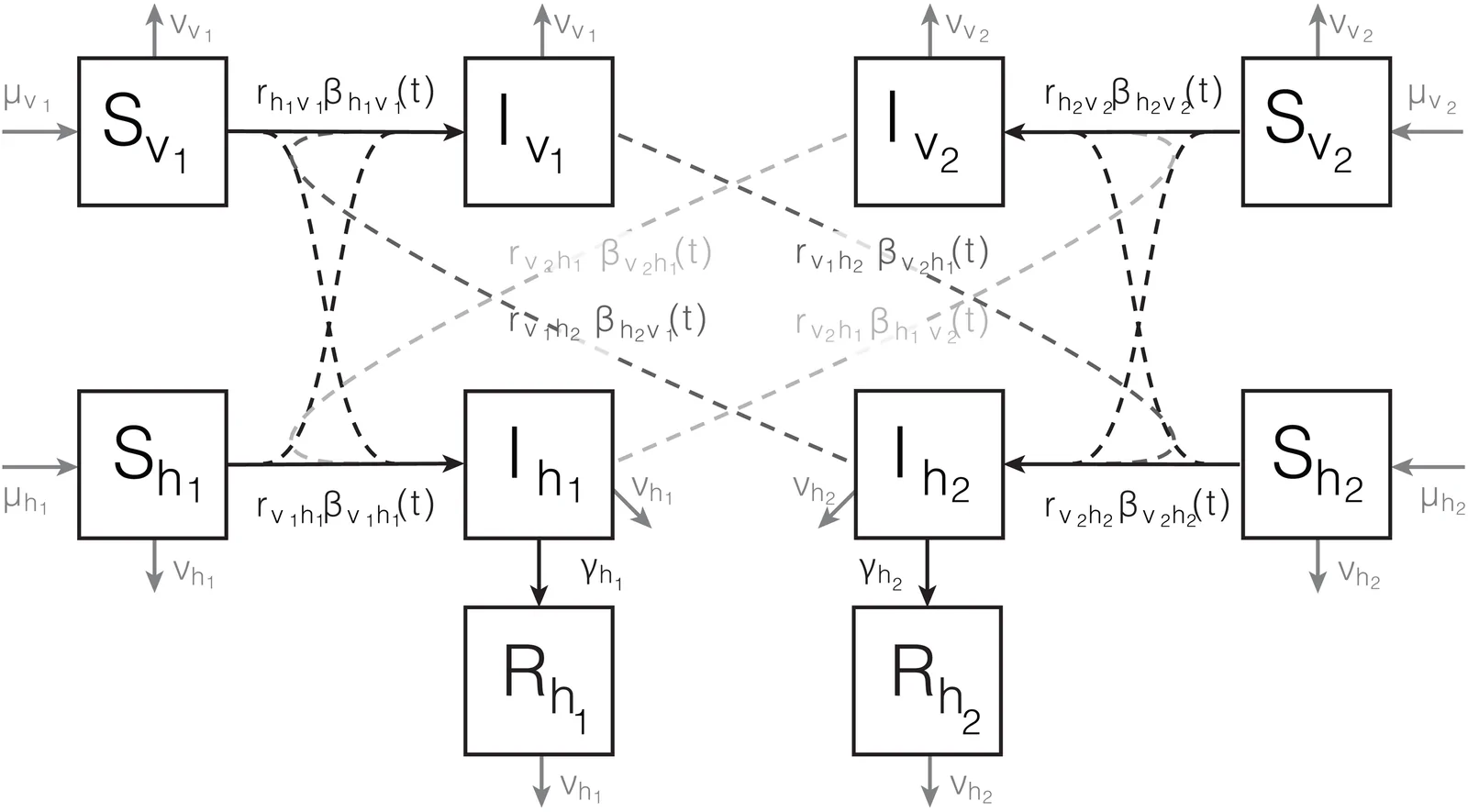
Diagram of the multi-host, multi-vector SIR model. The schematic shows two host species (distinguished by different shading) and two vector species, each preferentially feeding on one host species. Light gray arrows indicate cross-species biting (a vector feeding on its non-preferred host), whereas black arrows indicate a vector feeding on its primary host. Each transmission route is characterized by a biting rate, $r_{v_{j}h_{i}}$, between host *i* and vector *j*, and a probability of infection per bite (which may differ for host-to-vector vs. vector-to-host transmission and can vary seasonally). Infected primates recover at rate 1/γ, moving to the recovered class. Host and vector populations experience births and deaths (gray arrows) such that each population remains approximately constant in size. See text for additional details, S1 File, and Althouse et al. [3,55] for the full model equations.

To examine the effects of transmission-term formulation on multi-host, multi-vector disease dynamics, I utilize a flexible model developed previously [3,15,55], which explicitly tracks pathogen dynamics in both hosts and vectors as diagrammed in Fig 1. The model was originally formulated to examine the role of multiple primate species in shaping dengue virus isolations in southeastern Senegal. The basic model equations are given in S1 File. Here I present results not specific to any one pathogen, but for a general pathogen infecting multiple hosts and vectors, generalizable to *i* host species and *j* vector species.

Each host species is modeled with susceptible (*S*), infectious (*I*), and recovered (*R*) compartments, while each vector species has susceptible and infectious compartments (with no recovered stage for vectors). Hosts recover and acquire immunity at rate $1/\gamma_{i}$ (where $\gamma_{i}$ is the recovery rate for host *i*), whereas vectors do not recover from infection and remain infectious until they die. I assume host and vector population sizes are at demographic equilibrium, with births balancing deaths so that each host population $N_{h_{i}}$ and vector population $N_{v_{j}}$ stays approximately constant. For simplicity, I do not include any latent (exposed) infection class for hosts or vectors, effectively assuming negligible incubation periods (or that latency is subsumed into the transmission rates). All host–vector contacts occur via mass-action mixing, as determined by the biting rates $r_{v_{j}h_{i}}$.

**Fig 2:**
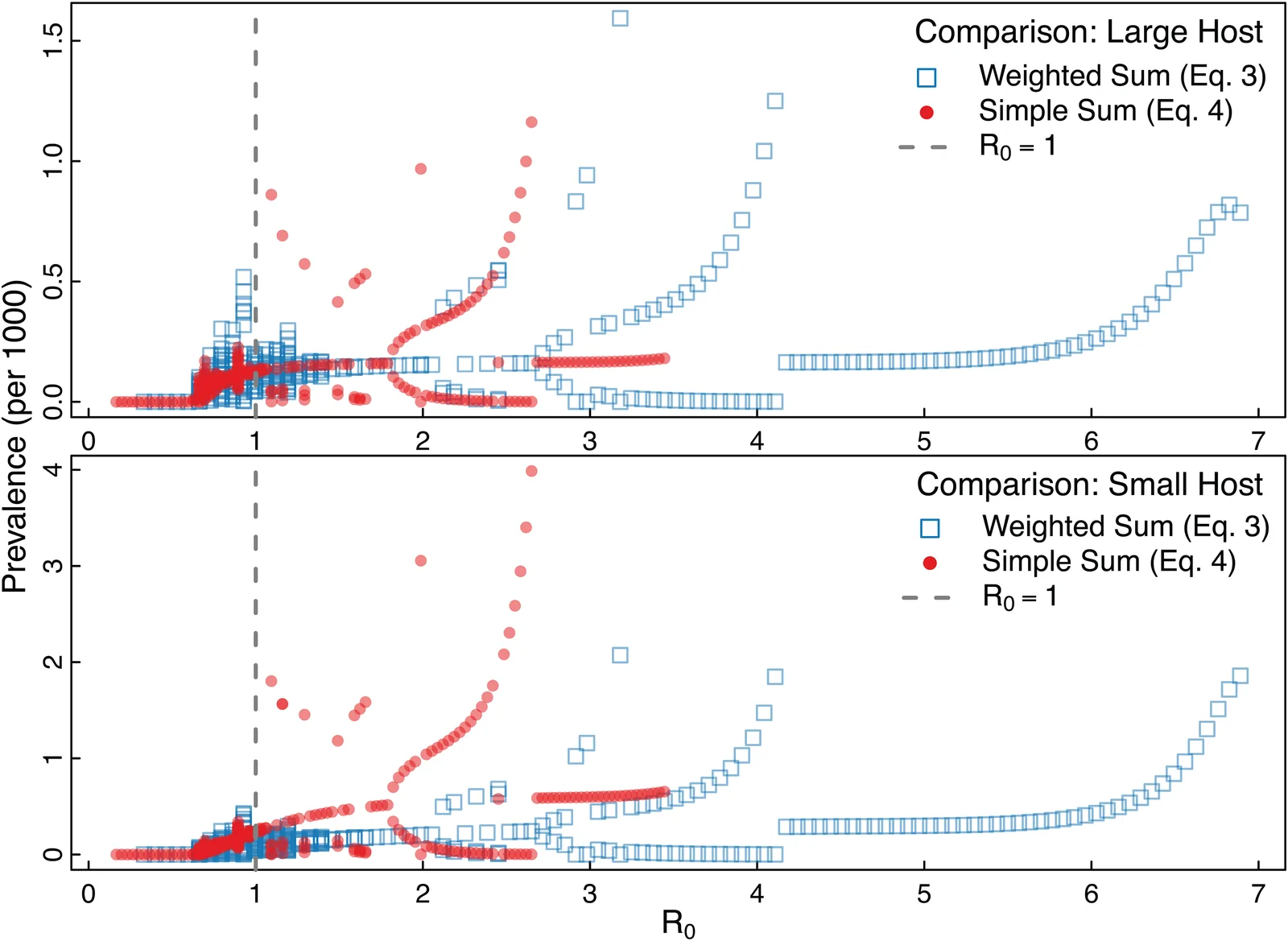
Transmission term formulation affects long-run infection prevalence. Host prevalence (per 1000 individuals) plotted against the basic reproduction number *R*_0_ for large primate (top) and small primate (bottom) hosts under two force-of-infection formulations. Squares denote the weighted (frequency-dependent, vector-limited) formulation (Eq. 4); circles denote the unweighted (density-dependent, host-limited) formulation (Eq. 5). The dashed vertical line marks *R*_0_ = 1. For each of 100 transmission probabilities β∈[0.025,0.520], points are annual samples from years 200–250 of one deterministic simulation with seasonal amplitude $c_{j}=0.05$; *R*_0_ is calculated for the corresponding mean transmission probability. Vertically aligned points therefore show temporal variation along a trajectory, not coexisting equilibria. The weighted formulation produces substantially higher prevalence and larger oscillation amplitudes at lower *R*_0_ values. *Parameters:* primary host biting rates $r_{m_{1}p_{1}}=r_{m_{2}p_{2}}=0.5$ bites per day; cross-species biting rates $r_{m_{2}p_{1}}=r_{m_{1}p_{2}}=0.001$ bites per day; host recovery rate $\gamma_{i}=1/4$ day^–1^ (4-day infectious period); large-host mortality $\mu_{p_{1}}=1/(60\times 365)$ day^–1^; small-host mortality $\mu_{p_{2}}=1/(15\times 365)$ day^–1^; vector mortality $\mu_{m_{j}}=1/7$ day^–1^; host populations $N_{p_{i}}=1,000$; vector populations $N_{m_{j}}=25,000$.

**Fig 3:**
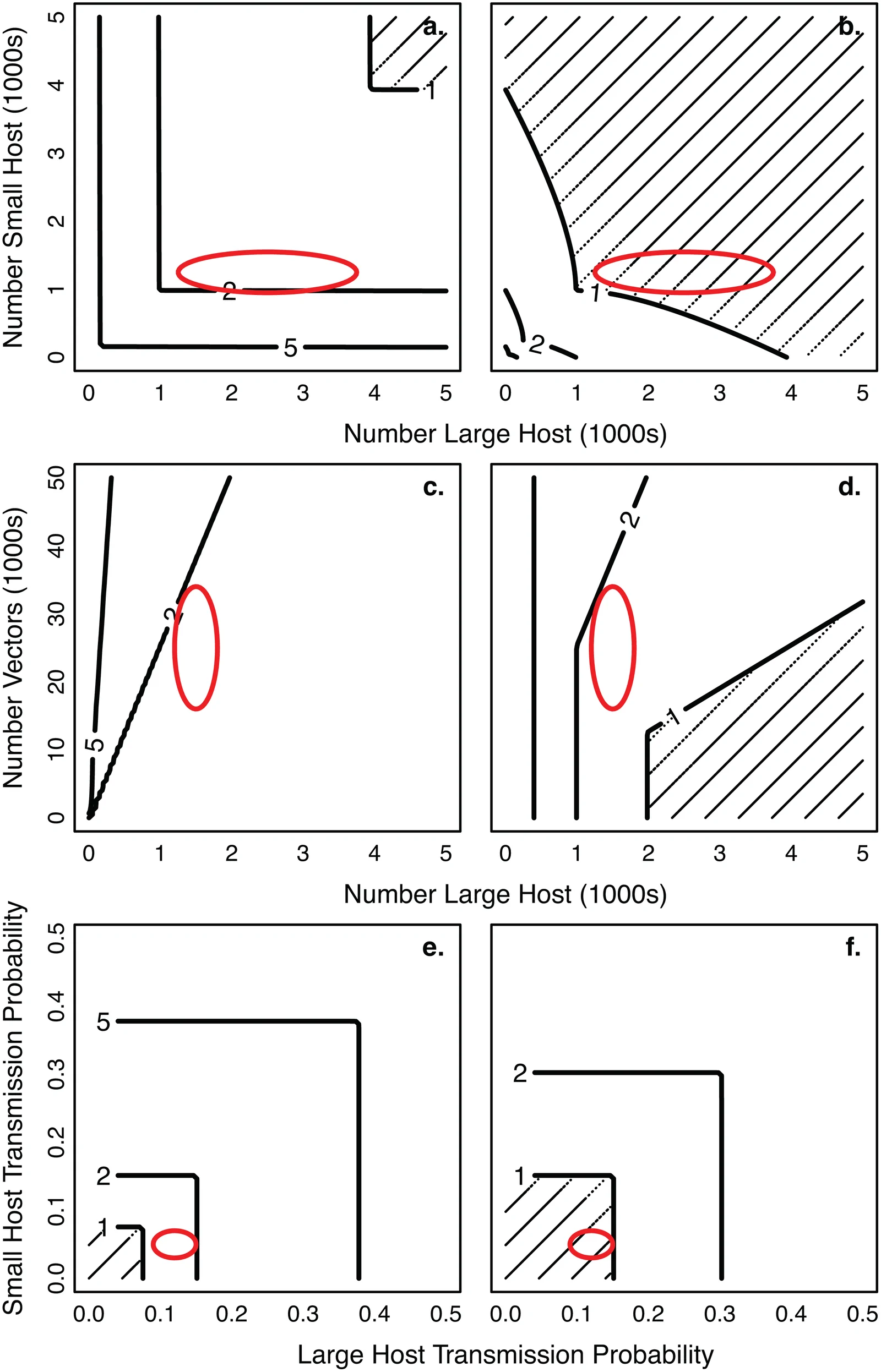
Divergent *R*_0_ outcomes under different transmission formulations. Heatmaps show the basic reproduction number, *R*_0_, for the two transmission-term formulations across a range of conditions. Left panels (**a**, **c**, **e**): weighted (frequency-dependent, vector-limited) formulation; right panels (**b**, **d**, **f**): unweighted (density-dependent, host-limited) formulation. Each pair of panels contrasts the two formulations while varying a particular set of parameters: panels **a** and **b** vary the numbers of large (low-birthrate) and small (high-birthrate) hosts; panels **c** and **d** vary the number of vectors and large hosts; panels **e** and **f** vary the transmission probabilities for large vs. small hosts. Hatched regions indicate *R*_0_ < 1 (no sustained transmission). Red circles denote plausible values for sylvatic DENV in Kédougou, Senegal. Notably, there are broad regions where identical parameter values produce *R*_0_ > 1 under one formulation but *R*_0_ < 1 under the other, leading to completely different predictions about outbreak potential. (All other parameters are as in Fig 2, with $b_{p_{i}m_{j}}=b_{m_{j}p_{i}}=0.15$.).

I model the force of infection on host *i* as


$$\begin{array}{c} \sum_{j}r_{v_{j}h_{i}}\;\beta_{v_{j}h_{i}}(t)\;\frac{I_{v_{j}}(t)}{N_{j}(t)}\;S_{h_{i}}(t), \end{array}$$ (1)

where $\beta_{v_{j}h_{i}}(t)$ is the seasonally forced probability of transmission from an infectious vector *j* to host *i* per bite, $I_{v_{j}}(t)$ is the number of infectious vectors of species *j*, and $S_{h_{i}}(t)$ is the number of susceptible hosts of species *i*. The general model permits seasonal forcing of the transmission probabilities via a cosine term with amplitude $c_{j}$ (see Section S1 in S1 File). The analytical *R*_0_ and center-manifold calculations set $c_{j}=0$ and apply to the autonomous system. The simulations summarized in Fig 2 instead use $c_{j}=0.05$ and show annual samples from years 200–250 of periodically forced trajectories. Those samples describe long-run prevalence and temporal variation, not fixed-point equilibria. Many arbovirus studies report species-specific *competence* parameters that modify the baseline probability of infection per bite. I therefore rewrite Eq. (1) as


$$\begin{array}{c} \lambda_{h_{i}}(t)=\sum_{j}r_{v_{j}h_{i}}\;\sigma_{h_{i}}\;\kappa_{v_{j}}\;\beta_{v_{j}h_{i}}(t)\;\frac{I_{v_{j}}(t)}{N_{j}(t)}\;S_{h_{i}}(t), \end{array}$$ (2)

where $\sigma_{h_{i}}$ is the intrinsic *host competence* (probability an exposed host becomes infectious) and $\kappa_{v_{j}}$ is the *vector competence* (probability an exposed vector becomes infectious). In the absence of species-specific data I set $\sigma_{h_{i}}=\kappa_{v_{j}}=1$, which is the baseline used in Figs 2 and 3.

The parameter $r_{v_{j}h_{i}}$ represents the biting preference weight of vector *j* for host species *i*, measured in units of per vector per day. The realized per-capita biting rate on host *i* depends on both this preference weight and the effective host availability (the denominator $N_{j}$). By construction $\sum_{i}r_{v_{j}h_{i}}=b_{j}$, the physiological biting capacity of vector *j* ($\approx 0.5\;{\text{day}}^{-1}$ for *Aedes*). Impose the standard ‘conservation‑of‑bites’ constraint $\sum_{i}r_{v_{j}h_{i}}N_{v_{j}}=\sum_{i}r_{h_{i}v_{j}}N_{h_{i}}$. Symmetry here means the *rate constants* are identical in the host‑to‑vector direction, i.e., $r_{v_{j}h_{i}}=r_{h_{i}v_{j}}$. All biting rates for the system can be encapsulated in a matrix **R** of biting rates:


$$\begin{array}{c} 𝐑=(\begin{array}{cccc} r_{h_{1}v_{1}} & r_{h_{2}v_{1}} & ... & r_{h_{i}v_{1}}\\ r_{h_{1}v_{2}} & r_{h_{2}v_{2}} & ... & r_{h_{i}v_{2}}\\ \vdots & \vdots & \ddots & \vdots \\ r_{h_{1}v_{j}} & r_{h_{2}v_{j}} & ... & r_{h_{i}v_{j}} \end{array})\;. \end{array}$$ (3)

In reality, there may be large heterogeneities in vector feeding behavior with no clear preferences in host selection (see Section 2.1). For notational convenience, I refer to the host species on which a given vector feeds most often as the “primary” host for that vector, and other hosts as “secondary.” I refer to the on-diagonal biting rates as the rates at which each vector feeds on its primary host, and the off-diagonal biting rates as the rates at which a vector feeds on secondary hosts. I assume off-diagonal biting events are less frequent than primary host bites.

The two alternative transmission formulations introduced above are distinguished by the definition of the denominator $N_{j}(t)$, which represents the effective number of hosts available to vector *j*. Extreme vector host specificity can be modeled as


$$\begin{array}{c} N_{j}(t)=\sum_{k}\left(\frac{r_{v_{j}h_{k}}}{\sum_{k}r_{v_{j}h_{k}}}\right)N_{h_{k}}\;, \end{array}$$ (4)

i.e., a weighted sum of the host populations, with weights proportional to the biting rates in **R**. As preferences for a given host change (increasing or decreasing $r_{v_{j}h_{k}}$ on host *k*), the population of hosts effectively encountered by the vector shifts accordingly. In the limit that $r_{v_{j}h_{k}}\to 0$ for a particular host *k*, that host contributes negligibly to $N_{j}(t)$. This formulation corresponds to an extremely efficient vector that “homes in” on a specific host and effectively ignores other hosts.

Alternatively, an inefficient (non-selective) vector can be modeled as


$$\begin{array}{c} N_{j}(t)=\sum_{k}N_{h_{k}}\;, \end{array}$$ (5)

an unweighted sum of all available hosts. In this case, for any set of biting rates $r_{v_{j}h_{k}}$ (bites per day), the pool of hosts accessible to the vector is the total host population, and the vector encounters hosts in proportion to their preference-weighted abundance ($r_{v_{j}h_{k}}N_{h_{k}}$). This corresponds to an opportunistic vector whose feeding pattern is driven primarily by host availability rather than innate preference.

These two denominators directly correspond to the biological assumptions about what limits mosquito-host contact, as outlined in the Introduction. The weighted formulation (Eq. 4) embodies the frequency-dependent, vector-limited framework in which mosquito search effort is the bottleneck: the total number of bites is constrained by vector physiology, and hosts compete for that fixed resource. The unweighted formulation (Eq. 5) embodies the density-dependent, host-limited framework in which host encounter probability is the bottleneck: each host presents an independent “target,” and aggregate biting can scale with total host availability. Note that in both formulations, the *relative allocation* of bites across host species is governed by the preference-weighted terms $r_{v_{j}h_{i}}N_{h_{i}}$ in the numerator; the denominator choice modifies the *scaling and nonlinear feedback* of per-vector exposure, not the host-allocation structure.

### 2.3 Model parameters and state variables

Table 1 provides a comprehensive listing of all state variables, parameters, their symbols, definitions, units, and default values used in the model. Complete model equations are provided in Section S1 of S1 File and in [55].

**Table 1:** Model state variables and parameters. Subscript *i* denotes host species ($i=1,...,n_{h}$); subscript *j* denotes vector species ($j=1,...,n_{v}$). Full model equations appear in Section S1 of S1 File. See also [55].

Symbol	Definition	Units	Default Range
*State Variables – Host Species i*			
$S_{h_{i}}(t)$	Susceptible hosts of species *i*	individuals	—
$I_{h_{i}}(t)$	Infectious hosts of species *i*	individuals	—
$R_{h_{i}}(t)$	Recovered hosts of species *i*	individuals	—
$N_{h_{i}}$	Total population, host species *i*	individuals	1,000–2,500
*State Variables – Vector Species j*			
$S_{v_{j}}(t)$	Susceptible vectors of species *j*	individuals	—
$I_{v_{j}}(t)$	Infectious vectors of species *j*	individuals	—
$N_{v_{j}}$	Total population, vector species *j*	individuals	25,000
*Transmission Parameters*			
$r_{v_{j}h_{i}}$	Biting preference rate, vector *j* on host *i*	day^–1^ vector^–1^	0.001–0.5
$\beta_{v_{j}h_{i}}(t)$	Transmission prob., vector *j* to host *i*	dimensionless	0.025–0.525
$\beta_{h_{i}v_{j}}(t)$	Transmission prob., host *i* to vector *j*	dimensionless	0.025–0.525
$\sigma_{h_{i}}$	Host competence	dimensionless	0.4–0.8
$\kappa_{v_{j}}$	Vector competence	dimensionless	0.15
$N_{j}(t)$	Effective host availability to vector *j*	individuals	Eq. 4/5
*Host Demographic & Recovery Parameters*			
$\mu_{h_{i}}$	Natural mortality, host species *i*	day^–1^	(15–60 yr)^–1^
$\alpha_{h_{i}}$	Disease-induced excess mortality rate, host species *i*	day^–1^	0
$\gamma_{i}$	Recovery rate, host species *i*	day^–1^	1/4
$b_{h_{i}}$	Birth rate, host species *i*	ind. day^–1^	$\mu_{h_{i}}N_{h_{i}}$
*Vector Demographic Parameters*			
$\mu_{v_{j}}$	Mortality rate, vector species *j*	day^–1^	1/7
$b_{v_{j}}$	Birth rate, vector species *j*	ind. day^–1^	$\mu_{v_{j}}N_{v_{j}}$
*Forcing & Derived Parameters*			
*a*(*t*)	Seasonal amplitude multiplier	dimensionless	1+0.3cos(2πt/365)
*R*_0_	Basic reproduction number	dimensionless	S1 File

### 2.4 Results: Contrasting dynamics under alternative FOI formulations

Importantly, these two forms of $N_{j}(t)$ lead to qualitative differences in the epidemiological dynamics of the system. Fig 2 compares the prevalence of infection in a host population under the two transmission formulations, in a system with two host species and two vector species. I observe a shift to higher infection prevalence at lower values of the transmission probability β under the weighted-sum formulation (Eq. 1). Thus, the more efficient vector can spread the pathogen more widely than the less efficient vector at lower transmission probabilities. Mechanistically, this difference arises because in the weighted formulation vectors concentrate most bites on the primary host species that sustains transmission, whereas in the unweighted formulation many bites are diverted to secondary hosts that contribute less to infection, effectively diluting overall transmission.

Fig 2 shows marked differences in long-run prevalence and oscillation amplitude between the two transmission formulations. Under the weighted-sum formulation (representing an efficient, host-targeting vector), annual samples from the final 50 years of each periodically forced simulation span higher prevalence values at lower transmission probabilities. The unweighted formulation (representing an opportunistic vector) produces lower prevalence and more strongly damped dynamics. Because the plotted points are repeated time samples from one trajectory at each parameter value, vertical point clouds show temporal variation rather than coexisting equilibria.

Analytical center-manifold calculations for the autonomous system (S1 File) give $a_{W}<0$, $a_{M}<0$, and *b* > 0. Thus both formulations undergo a forward (supercritical) transcritical bifurcation at their respective *R*_0_ = 1 thresholds. With disease-induced mortality absent, each host total $N_{h_{i}}=S_{h_{i}}+I_{h_{i}}+R_{h_{i}}$ is constant, so the weighted denominator $D_{j}=\sum_{k}\alpha_{kj}N_{h_{k}}$ is also constant. It therefore has no mixed derivatives with respect to infected compartments.

The time-series analyses demonstrate that the two formulations yield distinct long-run and transient behaviors. The weighted formulation is associated with sharper, high-amplitude oscillations in prevalence, reflecting rapid epidemic takeoffs followed by deep declines as susceptible hosts are depleted. The unweighted formulation produces more moderate, rapidly damped oscillations. These contrasting dynamics imply that vector feeding assumptions can alter both the outbreak threshold and the speed and intensity of epidemic trajectories, with important implications for public health interventions and surveillance strategies.

This pattern is echoed in Fig 3, which shows contour plots of *R*_0_ across various population sizes of large (low-birthrate) and small (high-birthrate) hosts, numbers of vectors, and transmission probabilities (an analytical expression for *R*_0_ is given in the Supporting Information of Althouse et al. [55], and code provided here as a supplement). For identical parameter values, there are regions where *R*_0_ < 1 (no transmission, indicated by hatching) under one formulation but *R*_0_ > 1 under the other. Thus, depending on the transmission-term formulation, one would arrive at conflicting epidemiological predictions for an otherwise identical scenario.

To isolate the role of total bite volume from that of denominator structure, I repeated the *R*_0_ comparison after rescaling the unweighted biting rates so that both formulations deliver the same total number of bites per vector at every parameter point (Section S3 in S1 File). This equal-bite normalization eliminates all *R*_0_ differences, confirming that the distinct landscapes in Fig 3 reflect the total-bite imbalance inherent in the two ecological hypotheses. With fixed host totals, the rescaling also makes the two ODE right-hand sides identical, providing a consistency check on the corrected local bifurcation calculation.

### 2.5 Field‑based parameterization for sylvatic DENV in the Kédougou, Senegal region

To anchor the theoretical landscape of Fig 3 in the real Kédougou (southeastern Senegal) sylvatic-DENV system, I converted published density surveys into absolute abundances for a 1,000 ${km}^{2}$ surveillance zone centered on 12.55° N, 12.25° W. Recent serosurveys in this region confirm active DENV-2 circulation among these primate hosts, with species-stratified forces of infection that are consistent with the differential-competence framework modeled here [8]. Table 2 lists the point estimates, and the derivations and uncertainty propagation are detailed below.

**(a) African green monkeys (*Chlorocebus sabaeus*).** Galat‑Luong and Galat [56] counted 1,100–6,200 individuals per 1,000 ${km}^{2}$ across six transects. I adopted the transect mean as $N_{\text{small}}=2.5\times 10^{3}$ and took half the range ($\pm 2.55\times 10^{3}$) as a conservative 95% uncertainty, giving an ellipse radius $r_{x}=1.25$ on the *x*‑axis of panel a.
**(b) Patas monkeys (*Erythrocebus patas*).** Distance sampling by Isbell and Young [57] produced 1.5±0.3 km^–2^ (mean ± SE). Scaling to 1,000 km^2^ yields $N_{\text{large}}=1.5\times 10^{3}\pm 3.0\times 10^{2}$, so $r_{y}=0.30$ in thousands for panels a, c.
**(c) Aedes vector population.** Human‑landing catches in gallery forest (1999–2011 rainy seasons) averaged 17.2±6.4 *Ae. furcifer/taylori* females per person‑night [10]. Assuming two peak biting hours and a 60% parity rate,

$$\begin{array}{c} N_{\text{vector}}=(17.2\pm 6.4)\times 1\;000\;\text{trap-nights}\approx (2.5\pm 0.9)\times 10^{4}, \end{array}$$

so $r_{y}=5$ on the 1,000‑vector scale of panel c. The mean agrees with CO_2_‑baited trap data in Diallo [58].
**(e)–(f) Host–vector transmission probabilities** $\beta_{\text{small}}$ **and** $\beta_{\text{large}}$. Hanley *et al.* [7] reported peak viremia of $5.9\log_{10}$ and $5.2\log_{10}$ copies ml^–1^ in African green and patas monkeys, respectively, and found that 15% of *Ae. furcifer* became infectious after a $5.5\log_{10}$ blood‑meal. Mapping viremia to host competence (σ) with a logistic fit gives $\sigma_{\text{green}}=0.80\pm 0.06$ and $\sigma_{\text{patas}}=0.40\pm 0.04$; vector competence is κ=0.15±0.02. Propagating uncertainties (β=σκ) yields $\beta_{\text{small}}=0.12\pm 0.03$ and $\beta_{\text{large}}=0.05\pm 0.02$; these define $r_{x}=0.03$ and $r_{y}=0.02$ in panel e.

**Table 2:** Mid–rainy‑season parameterization of the Kédougou sylvatic‑dengue focus. Point estimates correspond to the centers of the red ellipses in Fig 3; the “±” values give the semi‑axes ($r_{x},r_{y}$) used to visualize the 95 % uncertainty on each axis. AGM, African green monkey; patas, *Erythrocebus patas.*

Fig 3 panel	Quantity (symbol)	Point estimate	± (95 %)	Ref.
a	Small-host population, *N*_small_ (AGM)	$2.5\times 10^{3}$ ind.	$1.25\times 10^{3}$	[56]
b	Large-host population, *N*_large_ (patas)	$1.5\times 10^{3}$ ind	$0.3\times 10^{3}$	[57]
c	Total vector females, *N*_vector_	$2.5\times 10^{4}$	$0.9\times 10^{4}$	[10,58]
e	Small-host transmission prob. $\beta_{\text{small}}$	0.12	0.03	[7]
f	Large-host transmission prob. $\beta_{\text{large}}$	0.05	0.02	[7]

The resulting point estimates and their 95% uncertainty radii are plotted as red open ellipses in Fig 3, demonstrating that the empirical Kédougou system occupies a region where the two force‑of‑infection formulations yield markedly different epidemiological predictions.

## 3 Discussion and outlook

In this study, I have shown that the choice of force-of-infection (FOI) formulation in multi-host, multi-vector pathogen models has far-reaching implications. Two extreme formulations – one in which vectors efficiently target a specific host and another in which vectors feed opportunistically based on host availability – lead to qualitatively and quantitatively different predictions of disease spread. This discrepancy is especially pronounced in multi-host systems, where the relative abundance and competence of hosts interact with vector feeding behavior to influence transmission dynamics.

A major implication is that public health interventions could be misdirected if the incorrect FOI formulation is used. For example, zooprophylaxis strategies have been proposed to control West Nile virus by introducing or preserving non-competent host species that divert mosquito bites away from highly competent hosts [59]. However, if vectors are highly selective (i.e., following the efficient formulation), non-competent hosts may have little effect on biting patterns, thereby failing to reduce human infections. Conversely, if vectors are indeed opportunistic, increasing host diversity could lead to a dilution effect, thereby lowering the risk of an outbreak [22]. This underlines the necessity for careful empirical studies to characterize vector host-choice behavior before designing interventions.

Another significant implication is in the realm of spillover risk. In recent studies on dengue and yellow fever, it was noted that vector feeding preferences can drive the transition between sylvatic and urban transmission cycles [6,8,9]. If a vector is assumed to bite all hosts indiscriminately (as in the opportunistic formulation), models might underestimate the risk of spillover by diluting the effective contact rate with the primary reservoir. On the other hand, overestimating vector specificity (efficient formulation) could lead to an exaggerated estimation of the risk from a particular reservoir host. Accurately quantifying these preferences could determine whether control efforts should focus on reducing the density of a specific host or managing vector populations more generally.

Moreover, the implications extend to surveillance strategies. In a system where vectors show strong preference for a particular host (efficient formulation), surveillance efforts might be better targeted toward monitoring that host species for early outbreak signals. In contrast, if vectors are opportunistic, a broader surveillance approach encompassing multiple host species may be warranted. Chen et al. [27] illustrated that even subtle differences in host competence and vector feeding can shift outbreak thresholds substantially. Such insights are critical when attempting to predict the emergence of diseases such as dengue [55], Zika [15] or chikungunya [11], where the sylvatic cycle plays a significant role in maintaining pathogen reservoirs.

The corrected local analysis sharpens the interpretation of these results. In the absence of disease-induced mortality, host totals remain constant and both FOI denominators are fixed positive quantities. The center-manifold coefficient is therefore negative for both formulations, giving a forward transcritical bifurcation at each model’s *R*_0_ = 1 threshold. The present model does not support a lower eradication threshold or hysteresis. Its robust contribution is instead the demonstration that alternative ecological assumptions can substantially move the epidemic threshold and alter prevalence and oscillation amplitude. Recent seroprevalence data from sylvatic DENV-2 in Kédougou reveal species-stratified forces of infection and persistent infant seroconversion during inter-epidemic periods [8]; these patterns motivate direct estimation of host preference and competence but do not by themselves imply multiple equilibria.

A crucial validation of this framework comes from equal-bite normalization analysis (Section S3 in S1 File). When the unweighted FOI is normalized to match the total biting rate of the weighted FOI (by rescaling $r\to \tilde{r}=c_{j}\;r$ with $c_{j}=\bar{N}/D_{j}$), the two formulations become mathematically identical and the *R*_0_ surfaces coincide exactly (Fig A in S1 File). This establishes that the *R*_0_ differences shown in Fig 3 arise because, for the same biting-rate parameters, the two denominators deliver different total numbers of bites per vector — a real biological consequence of the two ecological hypotheses, not a mathematical artifact.

The equal-bite calculation also clarifies the dynamical comparison. With fixed host totals, substituting the rescaled biting rates makes the complete ODE right-hand sides identical, not only the next-generation matrices. The Jacobians, Hessians, and local bifurcation coefficients must then coincide. Thus the unnormalized models differ because their denominators imply different total contact rates for the same nominal biting parameters, rather than because one denominator introduces a distinct state-dependent feedback.

Seasonal forcing remains an important driver of sylvatic arbovirus transmission. Fig 2 shows that the weighted FOI can produce larger prevalence oscillations than the unweighted FOI for the same nominal biting and transmission parameters, potentially changing the timing and magnitude of spillover risk. The current simulations do not establish hysteresis: each vertical point cloud consists of repeated samples from a single forced trajectory. Demonstrating bistability in the non-autonomous model would require continuation of periodic orbits and simulations from multiple initial conditions. Blood-meal surveys and time-series data remain valuable because they can identify which FOI formulation and forcing response are empirically plausible.

Ultimately, our findings suggest that an intermediate or blended formulation of the FOI, which incorporates a parameter to modulate the degree of vector specificity, might be more realistic. Such a model could be operationalized in at least two complementary ways. First, following the approach explored here, one could introduce a parameter *s* (for specificity) that interpolates between the two denominator formulations:


$$\begin{array}{c} N_{j}(t)=s\cdot \left(\sum_{k}\left(\frac{r_{v_{j}h_{k}}}{\sum_{l}r_{v_{j}h_{l}}}\right)N_{h_{k}}\right)+(1-s)\cdot \left(\sum_{k}N_{h_{k}}\right). \end{array}$$

Here, *s* = 1 represents a purely specialist (weighted, vector-limited) model, while *s* = 0 represents the unweighted (density-dependent, host-limited) model.

Alternatively, one could fix the denominator (e.g., $N_{j}=\bar{N}=\sum_{k}N_{h_{k}}$) and instead vary the preference matrix **R** itself, ranging from fully opportunistic feeding (all entries equal, bites distributed by host abundance alone) to extreme specialization (diagonal matrix, each vector feeds only on its primary host). This approach would isolate the effect of feeding selectivity independently of the total biting rate constraint and could be particularly valuable for fitting models to empirical bloodmeal data. Combining both dimensions – varying the preference structure *and* the denominator scaling – could yield an even more comprehensive framework.

Both directions align with recent calls for more flexible modeling frameworks in multi-host disease ecology, where both amplification and dilution effects have been observed [22,26]. By fitting such hybrid models to data, researchers could quantify where on the specialist-generalist continuum a particular vector species lies, allowing for more accurate predictions of outbreak potential and the impact of interventions across diverse ecological settings.

The results presented here underscore the importance of aligning model assumptions with empirical reality. The consequences of misrepresenting vector host-choice behavior are not merely academic: they could result in substantial misestimations of outbreak risk and, consequently, in suboptimal public health interventions. As multi-host pathogens continue to emerge and re-emerge [6,9], ensuring that our models accurately reflect the underlying biological processes is not just a theoretical necessity, but a public health imperative.

## Supporting information

S1 FileSupporting information containing supplementary analyses of how alternative force of infection (FOI) formulations and transmission asymmetries influence model outcomes.**Fig A:** **Equal‑bite normalization makes** $R_{0}$ **surfaces identical.** Each panel pair mirrors the axes used in main-text Fig 3 (left panels: weighted [frequency-dependent] formulation; right panels: unweighted [density-dependent] formulation) but the opportunistic FOI now employs the renormalized biting matrix ${\tilde{r}}_{v_{j}h_{i}}$ (Eq 2), ensuring equal total biting rates. Hatching denotes $\left(R_{0}<1\right)$. After normalization the two $\left(R_{0}\right)$ surfaces become identical, confirming that the qualitative differences between the formulations in the main text arise from the biological assumptions about what constrains total biting—not from a mathematical artifact of unequal bite totals. **Fig B:** **Asymmetric transmission does not qualitatively alter the comparison between FOI formulations.** The panels reproduce the parameter sweeps in main-text Fig 3 with host-to-vector transmission one quarter of vector-to-host transmission, $\beta_{hv}=\frac{1}{4}\beta_{vh}$. Left panels show the weighted (frequency-dependent, vector-limited) formulation, and right panels show the unweighted (density-dependent, host-limited) formulation. Hatching denotes $R_{0}<1$, and red ellipses denote plausible parameter values for sylvatic DENV in Kédougou, Senegal.(PDF)
