# Supplementary material for: Ecological specialization in vectors alters transmission thresholds and endemic dynamics in multi-host, multi-vector systems
Source: PLoS Comput Biol. 2026 Sep 11;22(9):e1014754. doi: 10.1371/journal.pcbi.1014754 (PMC13592690; doi:10.1371/journal.pcbi.1014754)
Supplement: Supplementary file 1 [file pcbi.1014754.s001.pdf]

S1 File. Supporting Information for Ecological specialization in vectors alters transmission thresholds and endemic dynamics in multi-host, multi-vector systems

Benjamin M. Althouse

1 Model Equations

Our deterministic SIR model comes from Althouse et al. (2012), "Synchrony of sylvatic dengue isolations: a multi-host, multi-vector SIR model of dengue virus transmission in Senegal," *PLoS Neglected Tropical Diseases* 6(11): e1928. The original model equations are as follows, where S, I, and R denote the susceptible, infectious, and recovered classes of each species and v_j indicates mosquito species j and h_i indicates primate species i (all state variables and parameters are defined in Table 1 in the main text):

$$\begin{aligned}
S'_{v_j}(t) &= \mu_{v_j}(S_{v_j}(t)+I_{v_j}(t)) - \sum_i r_{v_jh_i}\sigma_{h_i}\,\kappa_{v_j}\beta_{h_iv_j}(t)I_{h_i}(t)S_{v_j}(t)/N_j(t) - \nu_{v_j}S_{v_j}(t)\\
I'_{v_j}(t) &= \sum_i r_{v_jh_i}\sigma_{h_i}\,\kappa_{v_j}\beta_{h_iv_j}(t)I_{h_i}(t)S_{v_j}(t)/N_j(t) - \nu_{v_j}I_{v_j}(t)\\
S'_{h_i}(t) &= \mu_{h_i}N_{h_i}(t) - \sum_j r_{v_jh_i}\sigma_{h_i}\,\kappa_{v_j}\beta_{v_jh_i}(t)I_{v_j}(t)S_{h_i}(t)/N_j(t) - \nu_{h_i}S_{h_i}(t)\\
I'_{h_i}(t) &= \iota N_{h_i}(t) + \sum_j r_{v_jh_i}\sigma_{h_i}\,\kappa_{v_j}\beta_{v_jh_i}(t)I_{v_j}(t)S_{h_i}(t)/N_j(t) - \gamma_{h_i}I_{h_i}(t) - \alpha_{h_i}I_{h_i}(t) - \nu_{h_i}I_{h_i}(t)\\
R'_{h_i}(t) &= \gamma_{h_i}I_{h_i}(t) - \nu_{h_i}R_{h_i}(t),
\end{aligned}$$

with

$$\begin{aligned}
\beta_{h_iv_j}(t) &= b_{h_iv_j}[1+c_j\cdot\cos(t*2\pi/365)]\\
\beta_{v_jh_i}(t) &= b_{v_jh_i}[1+c_j\cdot\cos(t*2\pi/365)]\\
N_{v_j} &= S_{v_j}+I_{v_j}\\
N_{h_i} &= S_{h_i}+I_{h_i}+R_{h_i}\\
N_j(t) &= \sum_k\left(\frac{r_{v_jh_k}}{\sum_k r_{v_jh_k}}\right)N_{h_k} \qquad \text{(weighted / specialist formulation, main text Eq. 4).}
\end{aligned}$$

For the constant-normalization (opportunistic) variant (main text Eq. 5), we instead set

$$N_j(t) \equiv \bar{N} = \sum_k N_{h_k},$$

the total host abundance. All other equations remain unchanged; the two formulations differ *only* in this denominator.

Note on seasonal forcing. The cosine term with amplitude c_j permits seasonal modulation of the transmission probabilities $\beta(t)$. The R_0 and local bifurcation calculations set $c_j = 0$, so they apply to the autonomous system. The simulations summarized in main-text Fig 2 instead used $c_j = 0.05$ and plotted annual samples from years 200–250. Those points describe long-run prevalence in a periodically forced system; they are not fixed-point equilibria or an equilibrium-continuation diagram.

2 Local bifurcation at the disease-free equilibrium

2.1 Recap of the infection subsystem

For each host species h_i ($i = 1, \dots, H$) and vector species v_j ($j = 1, \dots, V$), set the importation rate and disease-induced mortality to zero, as in the analyses in the main text. Host and vector totals are then constant. The infection and recovery subsystem is

$$\begin{aligned}
\dot{I}_{h_i} &= \lambda_{h_i}\, S_{h_i} - (\gamma_i + \mu_{h_i})\, I_{h_i},\\
\dot{R}_{h_i} &= \gamma_i I_{h_i} - \mu_{h_i} R_{h_i},\\
\dot{I}_{v_j} &= \lambda_{v_j}(t)\, S_{v_j} - \mu_{v_j}\, I_{v_j},
\end{aligned}$$

where $S_{h_i} = N_{h_i} - I_{h_i} - R_{h_i}$ and $S_{v_j} = N_{v_j} - I_{v_j}$ with fixed demographic totals N_{h_i}, N_{v_j}. The forces of infection (FOI) are

$$\begin{aligned}
\lambda^{\mathrm{W}}_{h_i} &= \sum_{j=1}^{V} r_{v_j h_i}\, \beta_{v_j h_i}\, \frac{I_{v_j}}{D_j}, & D_j &= \sum_{k=1}^{H} \alpha_{kj} N_{h_k},\\
\lambda^{\mathrm{M}}_{h_i} &= \sum_{j=1}^{V} r_{v_j h_i}\, \beta_{v_j h_i}\, \frac{I_{v_j}}{\bar{N}}, & \bar{N} &= \sum_{k=1}^{H} N_{h_k},\\
\lambda_{v_j} &= \sum_{k=1}^{H} r_{v_j h_k}\, \beta_{h_k v_j}\, \frac{I_{h_k}}{q_j}, & q_j &= D_j \text{ or } \bar{N}.
\end{aligned}$$

The crucial point is that both D_j and $\bar{N}$ are constants in this model. Infection redistributes each host total among S, I, and R; it does not change N_{h_i} when disease-induced mortality is absent.

2.2 Disease-free equilibrium and Jacobian

Let $X = (I_{h_1}, \dots, I_{h_H}, I_{v_1}, \dots, I_{v_V})^\top$. Write the system as $\dot{X} = f(X) = F(X) - V(X)$ with

$$F = \begin{pmatrix} \Lambda_H(I_V)\, S_H \\ \Lambda_V(I_H)\, S_V \end{pmatrix}, \qquad V = \begin{pmatrix} \Gamma\, I_H \\ G\, I_V \end{pmatrix},$$

$$\Gamma = \mathrm{diag}(\gamma_1 + \mu_{h_1}, \dots, \gamma_H + \mu_{h_H}), \quad G = \mathrm{diag}(\mu_{v_1}, \dots, \mu_{v_V}).$$

At the disease-free equilibrium (DFE) $X = 0$ we have $S_{h_i} = N_{h_i}$ and $S_{v_j} = N_{v_j}$.

Linearization and next-generation matrices. We obtain the matrices B and C by linearizing the infection subsystem at the DFE using the FOI expressions defined above. Each entry is the coefficient of the corresponding infected compartment in the linearized equations $\dot{I}_h = B\, I_v - \Gamma\, I_h$, $\dot{I}_v = C\, I_h - G\, I_v$. For the preference-weighted formulation the denominator is $q_j = D_j$; for the constant-normalization formulation it is $q_j = \bar{N}$.

The Jacobian at the DFE is

$$Df(0) = \begin{pmatrix} -\Gamma & B \\ C & -G \end{pmatrix},$$

with

$$B_{ij} = \frac{r_{v_j h_i} \beta_{v_j h_i} N_{h_i}}{q_j}, \qquad C_{ji} = \frac{r_{v_j h_i} \beta_{h_i v_j} N_{v_j}}{q_j}.$$

The next-generation matrix and reproduction number are therefore

$$K = \begin{pmatrix} 0 & BG^{-1} \\ C\Gamma^{-1} & 0 \end{pmatrix}, \qquad R_0 = \rho(K) = \sqrt{\rho(BG^{-1}C\Gamma^{-1})}.$$

For multiple hosts and vectors this spectral-radius expression should not, in general, be replaced by a simple sum over transmission routes.

2.3 Center-manifold setup

Introduce a scalar bifurcation parameter η that scales all transmission probabilities, and define $\eta = 0$ to be the value at which $R_0 = 1$. At that value the DFE has a simple zero eigenvalue. Let v and w be the right and left eigenvectors of $Df(0)$ associated with this eigenvalue, normalized so that $w^\top v = 1$.

The dynamics restricted to the one-dimensional center manifold are

$$\dot{z} = a\, z^2 + b\, \eta\, z,$$

where

$$a = \tfrac{1}{2}\, w^\top D^2 f(0)\,(v, v), \qquad b = w^\top D f_\eta(0)\, v.$$

The bifurcation is *forward* (super-critical) if $a < 0, b > 0$ and *backward* (sub-critical) if $a > 0, b > 0$.

2.4 Evaluation of a and b

At the simple threshold, irreducibility of the host–vector transmission network permits the right and left null eigenvectors to be oriented so that their infection components are non-negative, with $w^\top v = 1$.

Second derivatives. Because q_j is constant, the only nonzero second derivatives at the DFE come from susceptible depletion. For either formulation they are

$$\frac{\partial^2 f_{I_{h_i}}}{\partial I_{v_j} \partial I_{h_i}} = \frac{\partial^2 f_{I_{h_i}}}{\partial I_{v_j} \partial R_{h_i}} = -\frac{r_{v_j h_i} \beta_{v_j h_i}}{q_j}, \qquad \frac{\partial^2 f_{I_{v_j}}}{\partial I_{h_i} \partial I_{v_j}} = -\frac{r_{v_j h_i} \beta_{h_i v_j}}{q_j}.$$

All cross-host derivatives are zero. In particular, differentiating $D_j = \sum_k \alpha_{kj} N_{h_k}$ with respect to an infected compartment is incorrect when N_{h_k} is fixed.

Quadratic coefficient a. Every nonzero contribution to $a = \frac{1}{2} w^\top D^2 f(0)(v, v)$ is non-positive, and at least one is strictly negative in a transmitting system. Consequently,

$$a_{\mathrm{W}} < 0, \qquad a_{\mathrm{M}} < 0. \tag{1}$$

Linear coefficient b. Because increasing η increases every transmission term and the relevant components of v and w are non-negative, $b = w^\top F_0 v > 0$.

2.5 Bifurcation type

$$a_M < 0, \quad a_W < 0, \quad b > 0$$

$\Longrightarrow$ **forward (supercritical) transcritical bifurcations in both FOI formulations.**

The denominator changes the location of the threshold and the endemic prevalence above it, but a constant positive denominator does not create the nonlinear feedback required for a backward bifurcation.

2.6 Numerical verification

The reproducibility script `compute_bifurcation_coefficients.R` independently evaluates the Jacobian and Hessian by finite differences and by closed-form derivatives. Using its eigenvector and coefficient normalization, it gives

$$a_{\mathrm{M}} = a_{\mathrm{W}} = -1.138859 \times 10^{-4}, \qquad b_{\mathrm{M}} > 0, \quad b_{\mathrm{W}} > 0.$$

The finite-difference and analytical values of a agree to better than 10^{-12}. The magnitude of a normal-form coefficient depends on eigenvector and one-half conventions, but its negative sign and the forward direction do not.

3 Sensitivity to equal-bite normalization

Equal-bite renormalization of the opportunistic FOI

In the main text we showed that the weighted (specialist) and un-weighted (opportunistic) FOI denominators can yield sharply different R_0 landscapes (main-text Fig 3). Those calculations, however, allowed the specialist formulation to deliver more bites per vector than the opportunistic one whenever host abundances were unequal. Here we repeat the analysis with the *equal-bite* scaling of Eq. (2), ensuring that every vector species j supplies the *same* total number of bites per day in both models.

Define $\bar{N} = \sum_i N_{h_i}$ (total host abundance) and

$$D_j \;=\; \sum_k \frac{r_{v_j h_k}}{\sum_l r_{v_j h_l}}\, N_{h_k}$$

(the preference-weighted effective host population appearing in the denominator of the weighted FOI, main text Eq. 4). Main text Equations (4) and (5) differ only in this denominator, yet the choice changes the *total* number of bites each vector delivers. For vector species j the weighted formulation (main text Eq. 4) yields a total bite flux

$$B_j^{\mathrm{W}} = \sum_i r_{v_j h_i},$$

whereas the unweighted formulation (main text Eq. 5) gives $B_j^{\mathrm{M}} = \sum_i r_{v_j h_i} N_{h_i}/\bar{N}$. To guarantee a direct comparison we renormalize the biting matrix that appears in the un-weighted transmission term:

$$\tilde{r}_{v_j h_i} \;=\; c_j\, r_{v_j h_i}, \quad \text{where} \quad c_j \;=\; \frac{B_j^{\mathrm{W}}}{B_j^{\mathrm{M}}} = \frac{\bar{N}}{D_j}\,. \tag{2}$$

Multiplying every $r_{v_j h_i}$ by the scalar c_j (computed at each $(N_{h_1}, \ldots, N_{h_i})$ grid-point) restores $B_j^{\mathrm{M}} = B_j^{\mathrm{W}}$ while preserving the original preference ratios $r_{v_j h_i} : r_{v_j h_k}$. Fig A visualizes the effect: the two FOI formulations now differ *only* in their denominators, not in total biting rates.

Equal-bite normalization makes the two models identical. Substituting the normalized biting rates $\tilde{r}_{v_j h_i} = c_j r_{v_j h_i}$ into the un-weighted FOI confirms that the two formulations become algebraically identical:

$$\tilde{\lambda}_{h_i}^{\mathrm{M}} = \sum_j \tilde{r}_{v_j h_i} \beta_{v_j h_i} \frac{I_{v_j}}{\bar{N}} = \sum_j c_j r_{v_j h_i} \beta_{v_j h_i} \frac{I_{v_j}}{\bar{N}} = \sum_j r_{v_j h_i} \beta_{v_j h_i} \frac{I_{v_j}}{D_j} = \lambda_{h_i}^{\mathrm{W}},$$

where the third equality follows from $c_j = \bar{N}/D_j$. The same cancellation applies in both directions of transmission. Because every entry of the next-generation matrix $K = FV^{-1}$ is built from these same biting rates, the rescaling $r \to c_j\, r$ makes the two next-generation matrices identical entry by entry, so their spectral radii coincide: $R_0^{\mathrm{M,\,eq}} = R_0^{\mathrm{W}}$.

Direct numerical verification at five representative parameter points (covering the full range of host-abundance ratios used in the main text) confirms $R_0^{\mathrm{M,\,eq}}/R_0^{\mathrm{W}} = 1.000$ in every case. Fig A visualizes this result: the left (weighted) and right (normalized unweighted) R_0 surfaces are now identical.

This result has a precise interpretation: the R_0 difference between the two formulations reported in the main text (Fig 3) is entirely attributable to the fact that, for the same biting-rate parameters $r_{v_j h_i}$, the weighted and unweighted denominators deliver different total numbers of bites per vector. Once this total-bite imbalance is removed by rescaling $r \to c_j r$, the next-generation matrices become identical and the R_0 surfaces coincide. The R_0 comparison in the main text is nonetheless the biologically relevant one: given a single set of field-estimated biting preferences, the two ecological hypotheses about what constrains total biting (vector-limited vs. host-limited) yield different epidemiological predictions.

The equal-bite result also provides a useful consistency check on the local dynamics. With fixed host totals, both D_j and $\bar{N}$ are constants. Substitution of $\tilde{r}_{v_j h_i} = c_j r_{v_j h_i}$ therefore makes the two ODE

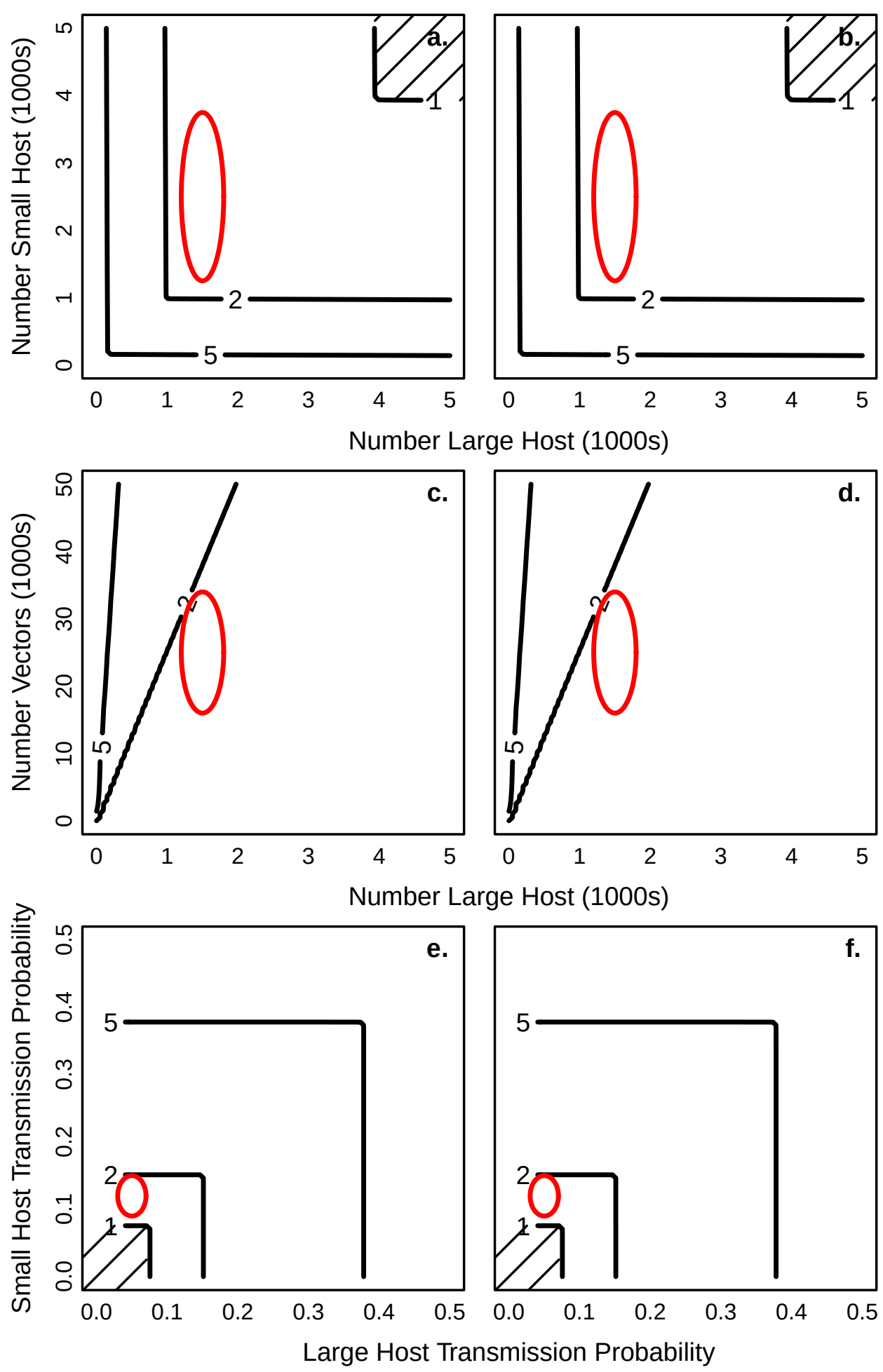

Fig A: **Equal-bite normalization makes R_0 surfaces identical.** Each panel pair mirrors the axes used in main-text Fig 3 (left panels: weighted [frequency-dependent] formulation; right panels: unweighted [density-dependent] formulation) but the opportunistic FOI now employs the renormalized biting matrix $\tilde{r}_{v_j h_i}$ (Eq. (2)), ensuring equal total biting rates. Hatching denotes $R_0 < 1$. After normalization the two R_0 surfaces become identical, confirming that the qualitative differences between the formulations in the main text arise from the biological assumptions about what constrains total biting — not from a mathematical artifact of unequal bite totals.

right-hand sides identical, not only their next-generation matrices. Their Jacobians, Hessians, and local bifurcation coefficients must consequently be identical under this normalization. As shown in Section 2 of S1 File, both unnormalized formulations also undergo a forward transcritical bifurcation at their respective $R_0 = 1$ thresholds.

4 Asymmetric transmission between hosts and vectors

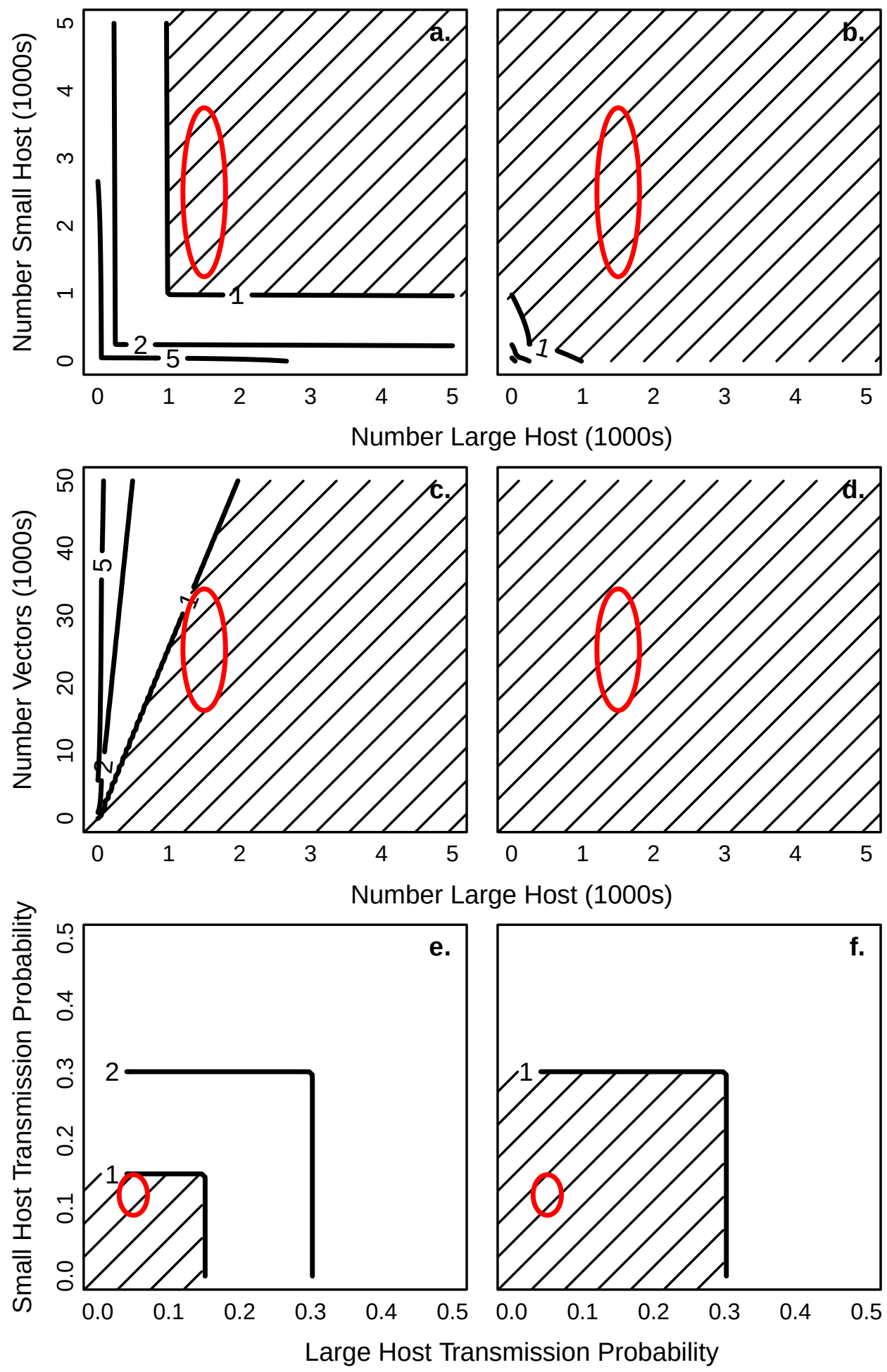

Fig B: **Asymmetric transmission does not qualitatively alter the comparison between FOI formulations.** The panels reproduce the parameter sweeps in main-text Fig 3 with host-to-vector transmission one quarter of vector-to-host transmission, $\beta_{hv} = \frac{1}{4}\beta_{vh}$. Left panels show the weighted (frequency-dependent, vector-limited) formulation, and right panels show the unweighted (density-dependent, host-limited) formulation. Hatching denotes $R_0 < 1$, and red ellipses denote plausible parameter values for sylvatic DENV in Kédougou, Senegal.
